# Effects of surgery start time on postoperative cortisol, inflammatory cytokines, and postoperative hospital day in hip surgery: Randomized controlled trial

**DOI:** 10.1097/MD.0000000000015820

**Published:** 2019-06-14

**Authors:** Young Suk Kwon, Ji Su Jang, Sung Mi Hwang, Hyunjin Tark, Jong Ho Kim, Jae Jun Lee

**Affiliations:** a Department of Anesthesiology and Pain Medicine, College of Medicine, Hallym University; b Department of Anesthesiology and Pain Medicine, College of Medicine, Kangwon National University; c Institute of New Frontier Research, College of Medicine, Hallym University, Chuncheon, South Korea.

**Keywords:** circadian rhythm, cortisol, hip, IL-6, IL-8, surgery start time

## Abstract

**Background:**

The aim of this study was to compare morning surgery (Group A), characterized by high cortisol levels, with afternoon surgery (Group B), characterized by low cortisol levels, with respect to cortisol, inflammatory cytokines (interleukin [IL]-6, IL-8), and postoperative hospital days (POHD) after hip surgery.

**Methods:**

The study was conducted in a single center, prospective, randomized (1:1) parallel group trial. Patients undergoing total hip replacement or hemiarthroplasty were randomly divided into two groups according to the surgery start time: 8 am (Group A) or 1–2 pm (Group B). Cortisol and cytokine levels were measured at 7:30 am on the day of surgery, before induction of anesthesia, and at 6, 12, 24, and 48 hours (h) after surgery. Visual analogue scale (VAS) and POHD were used to evaluate the clinical effect of surgery start time. VAS was measured at 6, 12, 24, and 48 h postoperatively, and POHD was measured at discharge.

**Results:**

In total, 44 patients completed the trial. The postoperative cortisol level was significantly different between the two groups. (24 h, *P* < .001; 48 h, *P* < .001). The percentage of patients whose level returned to the initial level was higher in Group B than in Group A (*P* < .001). Significant differences in IL-6 levels were observed between the two groups at 12, 24, and 48 h after surgery (*P* = .015; *P* = .005; *P* = .002), and in IL-8 levels at 12 and 24 h after surgery (*P* = .002, *P* < .001). There was no significant difference between the two groups in VAS and POHD. However, only three patients in Group A were inpatients for more than 3 weeks (*P* = .233).

**Conclusions:**

Afternoon surgery allowed for more rapid recovery of cortisol to the baseline level than morning surgery, and IL-6 and IL-8 were lower at 1–2 days postoperatively. The results of this study suggest that afternoon surgery may be considered in patients with postoperative delayed wound healing or inflammation because of the difference in cortisol, IL-6 and 8 in according to surgery start time.

Clinical trial registration number: NCT03076827 (ClinicalTRrial.gov).

## 1 Introduction

Cortisol is secreted by both the zona fasciculate and zona reticularis in response to stress. Cortisol levels follow a circadian rhythm under normal circumstances; cortisol secretion is highest at 8 am and then gradually decreases before reaching a minimum at midnight. However, cortisol levels increase in response to all types of stress, including trauma and infection.^[1]^ Surgical procedures cause stress and trauma, such that cortisol secretion markedly increases after surgery.^[2–5]^ Surgery and trauma can also affect the circadian rhythm of cortisol.^[6,7]^ We hypothesized that the circadian rhythm of cortisol might affect postoperative cortisol levels depending on the surgery start time.

The inflammatory response is an important prognostic indicator.^[8,9]^ Cytokines regulate the host response to infection, the immune response, inflammation, and trauma; pro-inflammatory cytokines exacerbate the disease state.^[8,10]^ Many studies have been performed on inflammatory cytokines after surgery.^[2–5]^ Some studies have measured changes in postoperative cortisol and cytokine levels over time,^[3–5]^ and one study reported an association between them in terms of such changes.^[2]^ No study has analyzed changes in cortisol and cytokines according to surgery start time. The aim of this study was to compare morning surgery, characterized by high cortisol levels, with afternoon surgery, characterized by low cortisol levels, with respect to cortisol, inflammatory cytokines and postoperative hospital days (POHD).

## 2 Materials and methods

### 2.1 Patient selection criteria

This study was conducted in a single-center, prospective, randomized (1:1) parallel group trial. The study took place at Sacred Heart hospital in Chuncheon, South Korea, from October 2015 to October 2016 and followed the principles of the Declaration of Helsinki. This study protocol was approved by Chuncheon Sacred Heart Hospital Institutional Review Board/Ethics Committee, and all patients provided informed consent before surgery. The clinical trial protocol was registered (ClinicalTRrial.gov NCT03076827). In total, 52 patients were assessed for eligibility. Eligible patients were those scheduled to undergo hip hemiarthroplasty or total hip replacement, with an American Society of Anesthesiologists (ASA) physical status grade of I–III and age of 20–100 years. Both men and women were included. The exclusion criteria were refusal to participate, body mass index > 30 kg/m^2^, and the presence of factors that affect the circadian rhythm of cortisol, such as a sleep disorder, a mental disorder, or current use of psychiatric medications.

### 2.2 Study design

In total, 48 patients participated in this study; four patients who refused to participate were excluded. The included patients were divided into morning-surgery (Group A) and afternoon-surgery (Group B) groups. The second author created a computerized permuted block schedule with random allocation to groups at a 1:1 ratio, and equal allocation to each block (block size = 4). The second author provided an opaque envelope containing allocation schedule to corresponding author. The research coordinator recruited participants, who were allocated sequentially on enrolment. The corresponding author assigned surgery start time to the patient to be scheduled for surgery according to randomized allocation schedule and delivered a sealed opaque envelope containing this information to the orthopedic office. The orthopedic surgeon confirmed the surgery start time 1 day before the surgery. Because of the nature of the research question, the study was not entirely blind, but the statistician, and the nurse who collected the blood, did not know which groups the patients belonged to. All patients underwent an 8 hours (h) fast before surgery. Anesthesia was started at 8 am in Group A and at 1–2 pm in Group B. We predicted that there would be differences in postoperative cortisol, interleukin (IL)-6 and IL-8 levels between the morning and afternoon surgery as primary outcomes. Postoperative pain and hospital stay data were analyzed additionally as secondary outcomes. In both groups, blood sampling was performed at 7:30 am, after induction of anesthesia, and at 6, 12, 24, and 48 h after surgery. A visual analogue scale (VAS; score: 0–10) was used to assess the degree of pain at 6, 12, 24, and 48 h after surgery. POHD and long-term inpatients (>3weeks) were determined when a patient was discharged from the hospital. All blood samples were obtained from peripheral veins. After induction of anesthesia, blood sampling was performed in the operating room, and other blood sampling was performed in the patient's room. The blood samples were sent to the lab on the day and the results of the analysis of the samples were given to the first author. The VAS pain scores were measured by the fourth author, and POHD was obtained through medical records at discharge.

All surgeries were performed by the same orthopedic surgeon and the surgical team. The patients were placed at the operating table on the lateral position during surgery. The incision centered on the posterior aspect of the greater trochanter of femur. Appropriate sized implants were inserted into the patient. The drain tube was inserted before the surgical wound was closed. After surgery, an abduction pillow was applied on bed to prevent the dislocation of the hip.

Anesthesia was performed in the same manner in both groups, and all patients received 0.2 mg of intramuscular glycopyrrolate 30 min before the surgery. After entering the operating theatre, patients were monitored using standard monitoring devices. Anesthesia was induced using 1.5 mg/kg propofol and 0.6 mg/kg rocuronium, and maintained with desflurane, nitrous oxide, and rocuronium. Opioids were not used during surgery. After surgery, muscle relaxation was reversed using glycopyrrolate and pyridostigmine. Patient-controlled analgesia (PCA) was used for appropriate pain management. PCA (loading dose = 5 μg/kg, continuous infusion rate = 3 μg/kg/h, bolus dose = 0.75 μg/kg, lock out interval = 15 min) was made up to 100 mL with 10 mg alfentanil. Postoperative pain management was performed by one same anesthesiologist. Additional analgesics were used when the postoperative VAS score was > 4 despite applying PCA. To reduce postoperative nausea and vomiting, ramosetron was administered 30 min before the end of surgery.

Postoperative management was performed by the same management team in both groups, and surgical wounds were treated at 24-h intervals. Postoperative wound management was performed by one same orthopedic surgeon. He monitored incision of patients daily for signs and symptoms of infection which many include: redness, swelling, warmth, drainage, increase in pain, or fever. Antibiotics were given depending on the patient's symptoms and signs. Patients began to use wheelchairs on the third day after surgery, and then began walking practice according to the patient's condition.

There were no changes of methods and trial outcomes after trial commencement.

### 2.3 Interleukin-6, interleukin-8, and cortisol levels

IL-6, IL-8, and cortisol levels were measured in blood taken from a peripheral vein at 7:30 am, after induction of anesthesia, and at 6, 12, 24, and 48 h after surgery. Plasma was separated by centrifugation and stored at −70°C until assay. The plasma IL-6, IL-8, and cortisol levels were determined using enzyme-linked immunosorbent assay kits (R&D Systems, Minneapolis, MN, USA). All assays were performed exactly as recommended by the manufacturer.

### 2.4 Statistics

Data are expressed as means and standard deviation (SD) or as numbers and percentages. The demographic data and patient characteristics of the two groups were analyzed using the *t*-test and the chi-square test. The primary outcome was a change in cortisol and interlerukin from 7:30 am on the day of surgery to 48 h postoperatively. Cortisol levels were compared between the two groups by repeated-measures analysis of variance (RM-ANOVA). The *t*-test was used as a post-hoc test for the RM-ANOVA to compare cortisol levels between the groups at each time point, and the Bonferroni method was applied. Because cortisol was measured six times, .008 (0.05/6) was taken as the corrected probability level. Kaplan–Meier curve analyses were conducted to calculate the percentages of patients whose cortisol and IL-6 levels returned to baseline at each time point after surgery. The percentages were compared between the groups using the log-rank test. Because the IL-6 and IL-8 levels were not normally distributed at any of the time points, the Mann–Whitney *U* test was used to compare the two groups. VAS and POHD were also analyzed with the Mann–Whitney *U* test. Fisher's exact test was used to compare the groups in terms of the number of inpatients who were hospitalized for more than 3 weeks. A probability of <.05 was considered statistically significant. Interim analysis was not carried out because the interim analysis method may be different from the final analysis method due to the small sample size. All data were analyzed using SPSS software (ver. 23.0; IBM Corp., Armonk, NY, USA). The sample size required was calculated using G∗power software (ver. 3.1; Universität Kiel, Kiel, Germany). Based on a pilot study (Group A mean = 111.6, Group B mean = 92.5, SD = 31.3, number of measurements = 6, total n = 10), 20 patients per group were required to detect a group difference in cortisol level according to RM-ANOVA (Cohen's effect size f(V) = .53, α = .05, 1 − β = .90) In total, we recruited 48 patients, given a predicted dropout rate of 20%.

## 3 Results

### 3.1 Study population and characteristics

**Figure 1:**
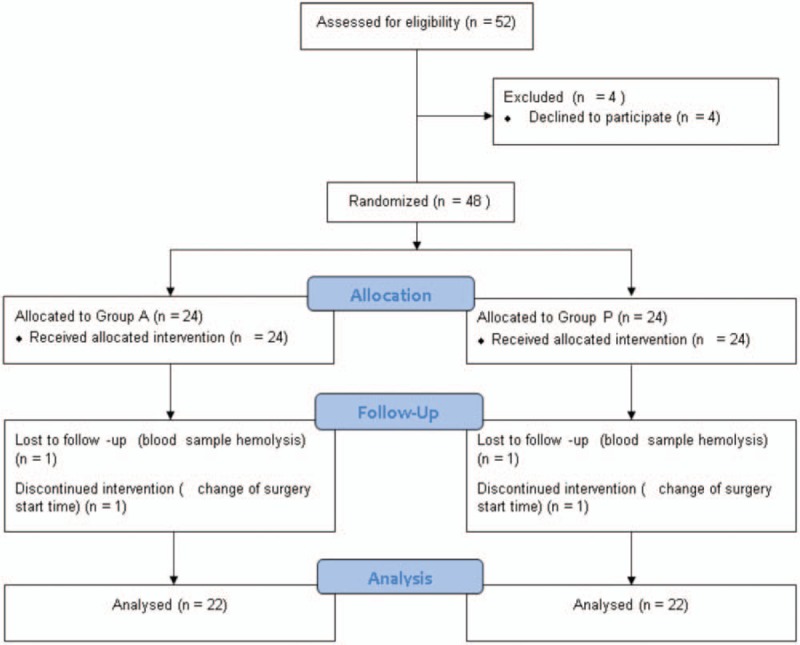
Flow chart. It shows Flow chart of participants in this randomised trial on the effects of surgery start time (and thus the circadian rhythm of cortisol) on postoperative interleukin-6, interleukin-8, and cortisol levels. Group A had a surgery start time of 8 am and Group B had a surgery start time of 1–2 pm.

Participants were recruited from October 1, 2015 to September 14, 2016. Although 52 patients were eligible, only 48 agreed to participate in the study and were thus included. The last participant underwent surgery on September 19, 2016, and was discharged on October 4, 2016. Of the 48 patients randomized, 44 completed the trial (Fig. 1). The morning surgery group (Group A) included 22 patients and the afternoon surgery group (Group B) included 22 patients. Two patients in Group A dropped out due to blood hemolysis (n = 1) and a change in surgery start time (n = 1). Two patients in Group B dropped out due to blood sample hemolysis (n = 1) and a change in surgery start time (n = 1).

No significant differences were observed between the two groups in terms of demographics, operation time, preoperative diagnosis, type of surgery, or ASA physical status (Table 1). The most common preoperative diagnosis was fracture in both groups. Half of the patients in each group were ASA physical status grade 2. Four (18.2%) patients in group A and five (22.7%) in group B were ASA physical status grade 3.

**Table 1:**
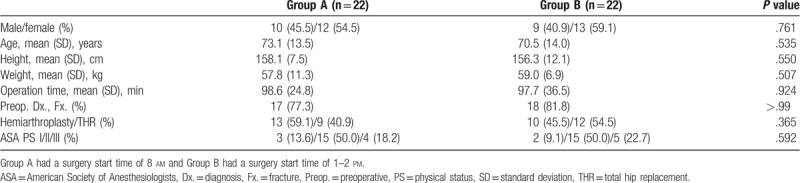
Demographic and clinical patient characteristics by Group.

### 3.2 Baseline data

The preoperative cortisol level was measured twice to confirm that there was no group difference in the baseline value, because the surgery start time varied and may be affected by the circadian rhythm (7:30 am, *P* = .832; before induction of anesthesia [BI], *P* = .674). IL-6 was also measured twice before surgery for the same reason, and there was no difference between the two groups (7:30 am, *P* = .133; BI, *P* = .1). IL-8 was measured at 7:30 am and before induction of anesthesia to confirm that there was no difference between the two groups before surgery (7:30 am, *P* = .799; BI, *P* = .973).

### 3.3 Laboratory and clinical outcomes

**Figure 2:**
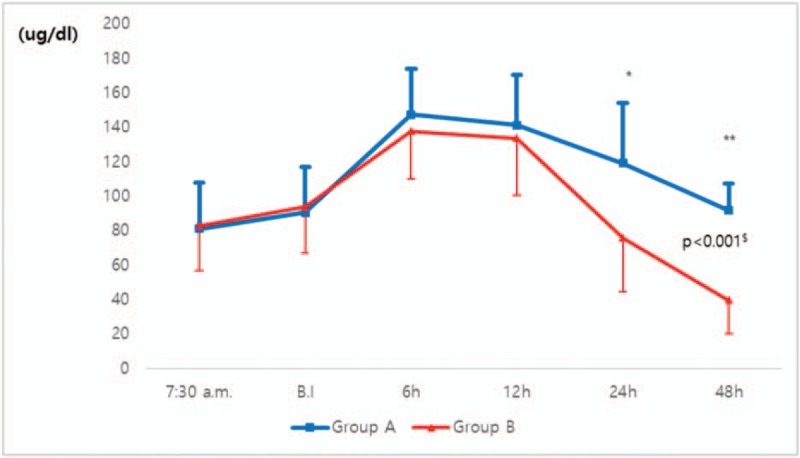
Comparison of cortisol levels between the morning and afternoon-surgery groups. Cortisol levels changed over time (*P* < .001, repeated measure ANOVA) and the degree of change differed between the two groups (^$^*P* < .001, repeated measures ANOVA). Post hoc test was corrected with Bonferroni's method (corrected probability of significance = .05/6 ≒ .008). There were differences between the two groups at 24 h (*P* < .001, *t*-test) and 48 h (*P* < .001, *t*-test) postoperatively. In 24 h after surgery, Group A (n = 22) was 119.2 μg/dL (SD = 31.3) and Group B (n = 22) was 75.8 μg/dL (SD = 35.1). The difference was 43.4 μg/dL (95% CI, 23.1–63.4, ^∗^*P* < .001). In 48 h after surgery, Group A was 91.7 μg/dL (SD = 19.4) and Group B was 39.7 μg/dL (SD = 15.7). The difference was 52.1 μg/dL (95% CI, 41.3–62.8, ^∗∗^*P* < .001). ANOVA = analysis of variance, BI = before anesthesia induction, CI = confidence interval, SD = standard deviation, 6–48 h, 6–48 h after surgery. Group A had a surgery start time of 8 am and Group B had a surgery start time of 1–2 pm.

Cortisol levels changed over time (*P* < .001) and the degree of change differed between the two groups (*P* < .001; Fig. 2). Cortisol increased up to 6 h after surgery and decreased thereafter in both groups. Post-hoc analysis revealed differences in cortisol levels between the two groups at 24 and 48 h postoperatively. At 24 h postoperatively, the cortisol level in Group A was 119.2 ± 31.3 μg/dL and that in Group B was 75.8 ± 35.1 μg/dL. At 48 h postoperatively, the respective levels were 91.7 ± 19.4 and 39.7 ± 15.7 μg/dL. The difference in cortisol level between the groups at 24 h postoperatively was 43.4 μg/dL (95% confidence interval [CI], 23.1–63.4, *P* < .001) and that at 48 h was 52.1 μg/dL (95% CI, 41.3–62.8, *P* < .001).

**Figure 3:**
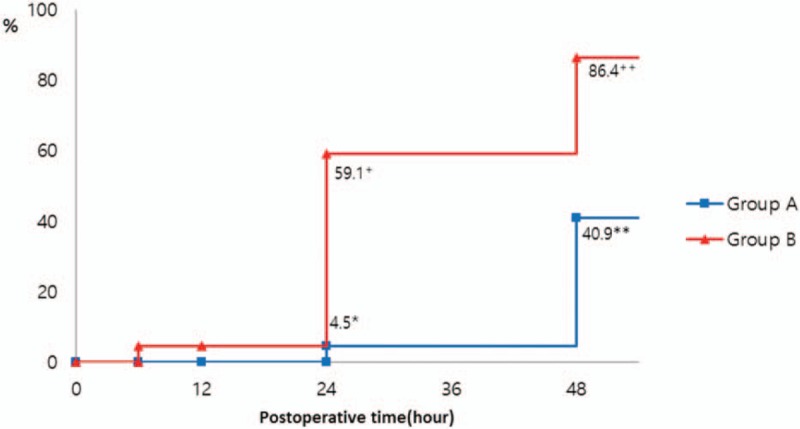
The percentage of patients returning to the initial level of cortisol. In Group A (n = 22), 4.5% (^∗^, n = 1) of the patients returned to the initial level at 24 h postoperatively and 40.9% (^∗∗^, n = 9 of the patients returned to the initial level at 48 h postoperatively. In Group B (n = 22), 59.1% (+, n = 13) of the patients returned to the initial level at 24 h postoperatively and 86.4% (++, n = 19 of the patients returned to the initial level at 48 h postoperatively (*P* < .001, Log-rank test). All percentages were analyzed by Kaplan–Meier curve analysis. Group A, percentage of return to initial values in the morning surgery group; Group B, percentage of return to initial values in the afternoon surgery group.

There was a significant difference between the two groups in the percentage of patients whose cortisol level returned to baseline after surgery, and a recovery to baseline levels was more likely during the earlier postoperative period in the afternoon surgery group (*P* < .001, Fig. 3). The percentage of patients with cortisol levels that returned to baseline was 4.5% in Group A and 40.9% in Group B at 24 h after surgery, and 59.1% and 86.4%, respectively, at 48 h after surgery.

**Figure 4:**
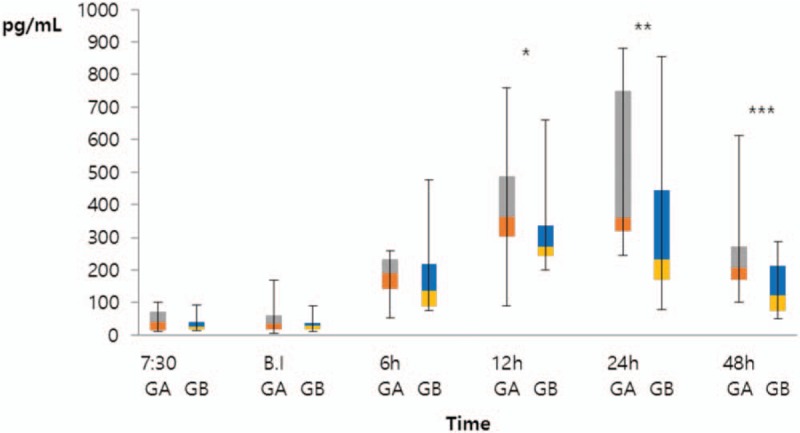
Comparison of interleukin-6 (IL-6) levels between the morning and afternoon-surgery groups. Group A (n = 22) and Group B (n = 22) were compared by the Mann–Whitney *U* test (12 h, ^∗^*P* = .015; 24 h, ^∗∗^*P* = .005; 48 h, ^∗∗∗^*P* = .002). At 12 h after surgery, the median IL-6 level of Group A was 363.4 pg/dL (interquartile range [IQR]: 303.0–485.2 pg/dL) and that of Group B was 270.9 pg/dL (IQR: 252.2–336.7 pg/dL). At 24 h after surgery, the median IL-6 level of Group A was 359.9 pg/dL (IQR: 320.3–749.6 pg/dL) and that of Group B was 232.8 pg/dL (IQR: 171.1–444.4 pg/dL). At 48 h after surgery, the median IL-6 level of Group A was 207.4 pg/dL (IQR: 169.9–271.3 pg/dL) and that of Group B was 123.2 pg/dL (IQR: 73.5–211.6 pg/dL). BI = before anesthetic induction, GA = Group A (morning surgery group), GB = Group B (afternoon surgery group), h = hours, IL = interleukin, IQR = interquartile range.

IL-6 increased until 24 h postoperatively and then decreased at 48 h postoperatively (Fig. 4). However, the IL-6 level was higher in the morning surgery group from 12 to 48 h postoperatively (12 h, *P* = .015; 24 h, *P* = .005; 48 h, *P* = .002). The median IL-6 level at 12 h postoperatively in Group A was 363.4 pg/dL (interquartile range [IQR]: 303.0–485.2 pg/dL) and that of Group B was 270.9 pg/dL (IQR: 252.2–336.7 pg/dL). The median IL-6 level of Group A at 24 h postoperatively was 359.9 pg/dL (IQR: 320.3–749.6 pg/dL) and that of Group B was 232.8 pg/dL (IQR: 171.1–444.4 pg/dL). The median IL-6 level of Group A at 48 h postoperatively was 207.4 pg/dL (IQR: 169.9–271.3 pg/dL) and that of Group B was 123.2 pg/dL (IQR: 73.5–211.6 pg/dL).

**Figure 5:**
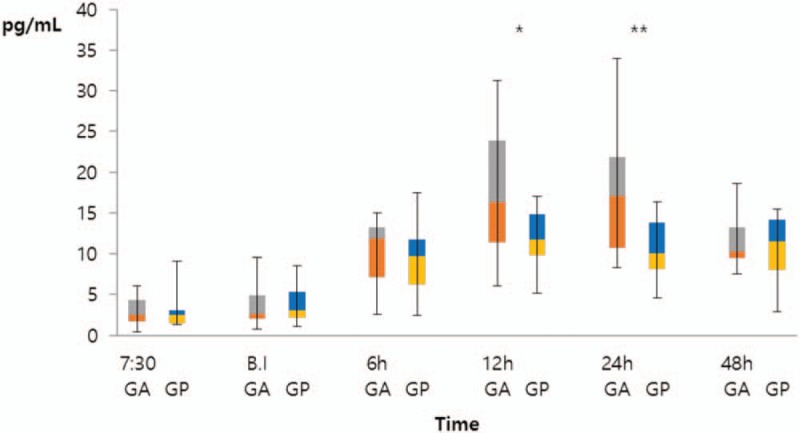
Comparison of Interleukin-8 (IL-8) levels between the morning and afternoon-surgery groups. Group A (n = 22) and Group B (n = 22) were compared by the Mann–Whitney *U* test (12 h, ^∗^*P* = .002; 24 h, ^∗∗^*P* < .001). At 12 h after surgery, the median IL-8 level of Group A was 16.3 pg/dL (interquartile range [IQR]: 11.4–23.8 pg/dL) and that of Group B was 11.9 pg/dL (IQR: 9.5–15.1 pg/dL). At 24 h after surgery, the median IL-8 level of Group A was 17.1 pg/dL (IQR: 10.8–21.9 pg/dL) and that of Group B was 10.1 pg/dL (IQR: 8.1–14.3 pg/dL). BI = before anesthetic induction, GA = Group A (morning surgery group), GB = Group B (afternoon surgery group), h = hours, IL = interleukin, IQR = interquartile range.

IL-8 increased in both groups up to 12 h postoperatively, but significant group differences were observed in IL-8 levels at 12 and 24 h after surgery (*P* = .002 and *P* < .001, respectively; Fig. 5). The median IL-8 level of Group A at 12 h postoperatively was 16.3 pg/dL (IQR: 11.4–23.8 pg/dL) and that of Group B was 11.9 pg/dL (IQR: 9.5–15.1 pg/dL). The median IL-8 level of Group A at 24 h postoperatively was 17.1 pg/dL (IQR: 10.8–21.9 pg/dL) and that of Group B was 10.1 pg/dL (IQR: 8.1–14.3 pg/dL). However, no significant difference was observed between the two groups at 48 h postoperatively.

No significant differences were observed between the two groups in the mean VAS score at any time point (6 h, *P* = .217; 12 h, *P* = .764; 24 h, *P* = .774; 48 h, *P* = .174), and the score declined over time in both groups (Table 2).

**Table 2:**
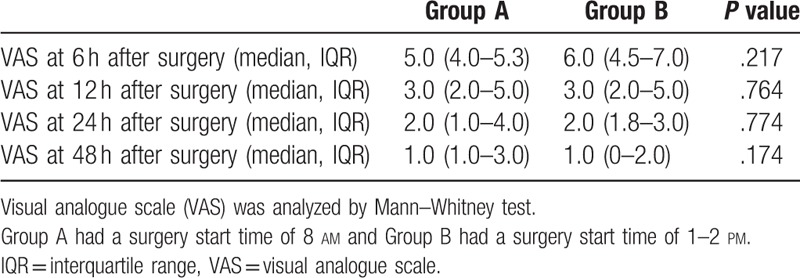
Comparison of visual analogue scale between the morning and afternoon-surgery groups according to the measurement time point after surgery.

No significant difference in POHD was observed between the two groups (*P* = .713; Table 3). The median POHD of Group A was 13.5 days (IQR: 12.0–16.8 days) and that of Group B was 13.0 days (IQR: 12.0–16.0 days). Based on previous studies on the relationship between POHD and patient safety, hospitalization for >3 weeks was used as the cut-off for subgroup analysis. No significant group difference was observed in the number of inpatients who were hospitalized for more than 3 weeks (*P* = .233, Table 3). There were three long-term patients in Group A but we could not calculate odds ratios because there was no long-term inpatient in the afternoon surgery group. There was no harm due to difference of surgery start time.

**Table 3:**
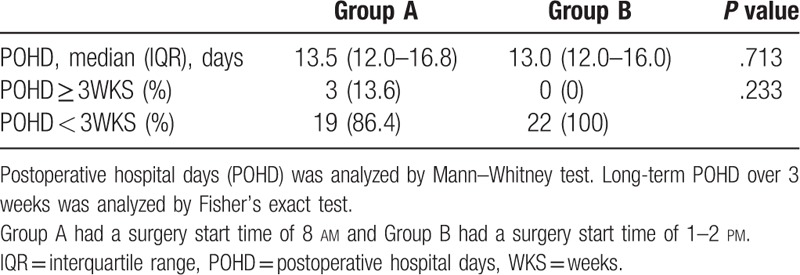
Comparison of postoperative hospital stay between morning and afternoon surgery group and comparison of the number of long-term hospitalized patients over 3 weeks.

## 4 Discussion

The cortisol level usually decreases in the afternoon. However, there were no differences in pre-operative (7:30 am and before anesthetic induction) cortisol levels due to differences in the start time of surgery in this study. Cortisol recovery to pre-operative level was faster in the afternoon surgery than in the morning surgery group. A difference in cortisol level between the two groups became apparent at 24 h postoperatively, and the percentage of patients in whom the cortisol level returned to baseline was lower in Group A. IL-6 levels started to increase rapidly after surgery and peaked at 12 h postoperatively. The IL-6 levels of the two groups showed significant differences at 12, 24, and 48 h after surgery, and were higher in Group A than in Group B. The IL-8 levels of the two groups were significantly different at 12 and 24 h after surgery, and were higher in Group A than in Group B. Patients with more than 3 weeks of hospitalization were only seen in group A.

In our study, postoperative cortisol abruptly increased and then gradually decreased, similar to previous studies. In some studies, cortisol recovered to baseline after 1 day, similar to that of the afternoon surgery group in this study.^[2,4,5]^ Postoperative cortisol recovered to the baseline level on the second day after surgery without PCA,^[5]^ as in the morning surgery group in our study. When changes in IL-6 levels over time were compared to changes in cortisol levels, a slower and delayed decrease in the former after reaching a peak was evident. These results were similar to those of previous studies,^[2,3,5]^ in which changes in cortisol occurred before increases in IL-6, suggesting that changes in systemic IL-6 levels are not responsible for changes in cortisol levels, at least initially.^[10]^ A median of 8 days of hospitalization is normal after hip surgery, and the hospitalization usually does not exceed 2 weeks. Age > 70 years, ASA physical status 3 and a long incision may increase the hospital stay.^[11]^ The median POHD in this study was long at about 13 days possibly due to the high average age of the patients (>70 years), the inclusion of nine patients with ASA physical status 3, and of cases of total hip replacement and hemi-arthroplasty with a long incision. Kelz et al reported that late surgery is associated with fatigue in medical staff due to their being fewer shift workers.^[12]^ However, in this study, the afternoon surgery was performed around 1 pm and usually ended before 4 pm. The number of medical staff on shift is not lower and at this time so post-operative management was not significantly different from that after the morning surgery. The time to the beginning and end of the afternoon surgery is not when the fatigue is accumulated due to lack of sleep. In previous studies,^[13,14]^ 1.5 to 3 h of sleep per day was defined as temporal sleep deprivation, but in this study, an orthopedic surgeon did not have such a pre-operative sleep deprivation.

Interestingly, there was no difference in cortisol levels according to differences in the time to induce anesthesia. It may be because the patients had fractures or necrosis of femoral head before surgery in this study. In this study, morning surgery was associated with more sustained increases in cortisol levels. If a high cortisol level persists, muscle wasting and downregulation of collagen synthesis may occur.^[15,16]^ High cortisol levels are associated with delayed wound healing and increased mortality.^[17–19]^ Stress-induced elevations in glucocorticoids can transiently suppress the production of inflammatory cytokines^[19]^ and elevate blood sugar levels via synergistic actions with other hormones,^[20]^ which is associated with delayed wound healing.^[21]^ IL-6 is rapidly released after injury due to an immune response and gradually decreases over several days. Its effects include promotion of the maturation, differentiation and activation of immune cells, in synergy with other mediators.^[22–24]^ Thus, IL-6 plays an important role in tissue damage and inflammatory responses.^[25]^ IL-8 is an important inflammatory cytokine that is produced after injury or surgery and plays an important role in neutrophil recruitment and degranulation.^[26,27]^ Neutrophil granulocytes are the major target cells of IL-8, but a relatively wide range of cells (endothelial cells, macrophages, mast cells, and keratinocytes) respond to chemokines. IL-8 can induce angiogenesis after surgery.^[28]^ Increased cortisol, IL-6 and IL-8 and a prolonged inflammatory response may cause tissue damage and delay recovery. No significant group difference was observed in the number of long-term (>3 weeks) hospitalizations; there were three such patients in Group A, and their cortisol levels did not return to initial levels over 48 h. If the hospital stay is 50% longer than expected, it may compromise patient safety.^[29]^ The expected hospital stay after hip surgery is less than 14 days;^[11]^ 3 weeks is more than 50% of that amount of time. Inappropriate pain management can affect most bodily systems and lead to progression of complications and prolonged hospitalization.^[30,31]^ We predicted that pain was one of the reasons for delayed discharge, but there was no difference between the two groups in VAS pain scores; this issue requires further study.

The strengths of our trial include its prospective, randomized design, used to evaluate the changes in and effects of postoperative cortisol, IL-6 and IL-8 levels according to surgery start time. However, this study also had some limitations. First, the sleep cycle can change depending on the type of surgery and the degree of pain,^[32,33]^ which can in turn affect cortisol levels, autonomic nerve stimulation and the levels of some cytokines.^[34]^ However, we did not assess changes in the sleep cycle after surgery. Second, if blood sampling had been performed at one more time point (i.e., at 36 h after surgery), we may have been able to demonstrate a benefit of afternoon surgery more clearly; 24 and 48 h after surgery correspond to noon for the morning surgery group and 4–5 pm for the afternoon surgery group. At this time point, cortisol is high after morning surgery in accordance with the circadian rhythm. In contrast, 12 and 36 h after surgery correspond to midnight for morning surgery and 4–5 am for the afternoon surgery group. At this time point, cortisol is high in afternoon surgery group in accordance with the circadian rhythm. Third, the possibility of unintentional bias could not be ruled out because the trial was not entirely blind. Forth, if the sample size was larger and the VAS pain scores were collected over a longer period, it could be improved in the analysis of the relationship between the start time of operation and long-term inpatient.

In conclusion, cortisol, IL-6, and IL-8 increased after surgery, but these changes were transient and the levels showed recovery pattern over 1–2 days after surgery. Afternoon surgery was associated with a more rapid recovery to the baseline cortisol level versus morning surgery, and IL-6 and IL-8 levels were lower at 1–2 days postoperatively. Differences in the changes in these levels after surgery did not cause major problems. Nevertheless, they can exert synergistic effects with other mediators or poor patient condition. The results of this study suggest that afternoon surgery may be considered in patients with postoperative delayed wound healing or inflammation because of the difference in cortisol, IL-6 and 8 in according to surgery start time.

## Acknowledgment

This research was supported by the Bio & Medical Technology Development Program of the National Research Foundation (NRF) funded by the Korean government (MSIT). (NRF-2017M3A9E8049714, 2017M3A9E8033205), South Korea.

## Author contributions

**Conceptualization:** Young Suk Kwon, Jae Jun Lee.

**Data curation:** Ji Su Jang, Sung Mi Hwang MD, Hyunjin Tark, Jong Ho Kim.

**Formal analysis:** Young Suk Kwon.

**Investigation:** Hyunjin Tark, Jong Ho Kim.

**Methodology:** Ji Su Jang.

**Supervision:** Sung Mi Hwang MD, Jae Jun Lee.

**Validation:** Jae Jun Lee.

**Writing – original draft:** Young Suk Kwon.

**Writing – review & editing:** Young Suk Kwon, Jae Jun Lee.
